# The multifaceted roles of matrix metalloproteinases in lung cancer

**DOI:** 10.3389/fonc.2023.1195426

**Published:** 2023-09-12

**Authors:** Cui Wei

**Affiliations:** Department of Emergency, The Third Hospital of Changsha, Changsha, China

**Keywords:** MMP, lung cancer, tumor immunity, drug resistance, MMP polymorphisms

## Abstract

**Background:**

Though the matrix metalloproteinases (MMPs) are widely investigated in lung cancer (LC), however, almost no review systematically clarify their multi-faced roles in LC.

**Methods:**

We investigated the expression of MMPs and their effects on survival of patients with LC, the resistance mechanisms of MMPs in anti-tumor therapy, the regulatory networks of MMPs involved, the function of MMPs inducing CSCLs, MMPs-related tumor immunity, and effects of MMP polymorphisms on risk of LC.

**Results:**

High expression of MMPs was mainly related to poor survival, high clinical stages and cancer metastasis. Role of MMPs in LC are multi-faced. MMPs are involved in drug resistance, induced CSCLs, participated in tumor immunity. Besides, MMPs polymorphisms may increase risk of LC.

**Conclusions:**

MMPs might be promising targets to restore the anti-tumor immune response and enhance the killing function of nature immune cells in LC.

## Introduction

The matrix metalloproteinases (MMPs) are a group of zinc-containing endopeptidases which are widely involved in extracellular matrix (ECM) degradation, tumor cell proliferation and invasion, cell differentiation and apoptosis, cancer development, immune response, et al (1, 2). MMPs family contains 28 members, and at least 23 of them are expressed in human tissues (3). MMPs show highly homologous structures. Based on their structural domains and substrates, 23 MMPs expressed in human tissues are mainly classified into six subgroups: collagenases (MMP-1, MMP-8 and MMP-13); gelatinases (MMP-2 and MMP-9); stromelysins (MMP-3, MMP-10 and MMP-11); matrilysins (MMP-7 and MMP-26); membrane-type (MT) MMPs (MMP-14, MMP-15, MMP-16, MMP-17, MMP-24, and MMP-25); and other MMPs (MMP-12, MMP-19, MMP-20, MMP21, MMP-23, MMP-27, MMP-28) (**Table 1**) (3–5). MMPs exert various functions in both physiological and pathological conditions. Under physiological conditions, MMPs are mainly involved in cell apoptosis, embryogenesis, immune response, morphogenesis, tissue remodeling, tooth enamel formation, wound healing, angiogenesis, et al (**Table 1**) (3). Under pathological conditions, MMPs contributes to chronic venous disease, fibrotic disorder, inflammation, lung and liver disease, viral infection, cancer development, et al (**Table 1**) (3).

**Table 1:** Classification, tissue distribution and functions of MMPs.

MMP	Human chromosome	Tissue distribution (Sorted by expression level from high to low)	Degradable ingredients	Functions under physiological conditions	Functions under pathological conditions
Collagenases					
MMP-1	11q22.2	Gall bladder, appendix, urinary biadder, endometrium, samll intestine, et al.	ECM, interstitial collagens I, II, and III.	Immune response, wound healing.	Fibrotic disorder (lung fibrosis, liver fibrosis), inflammation (increased cytokines), cardiovascular (atherosclerosis, aneurysm), cancer development.
MMP-8	11q22.3	Bone marrow, endometrium.	ECM, interstitial collagens I, II, and III.	Wound healing.	Inflammation (increased cytokines).
MMP-13	11q22.2	Urinary biadder, bone marrow, appendix, duodenum, testis, fat.	ECM, interstitial collagens, including types I, II, and III.	Tissue remodeling, Immune response.	Lung disease (asthma, COPD), osteoarthritis, viral infection (adenovirus, influenza).
Gelatinases					
MMP-2	16q12.2	Gall bladder, urinary bladder, endometrium, ovary, placenta.	ECM, gelatinase A, type IV collagenase.	Angiogenesis.	Viral infection (adenovirus, influenza), cardiovascular (atherosclerosis, aneurysm), chronic venous disease (varicose veins, venous leg ulcer), inflammation (increased cytokines) , EMT, cancer development.
MMP-9	20q13.12	Bone marrow, lymph node, appendix, spleen, urinary bladder.	ECM, type IV and V collagens.	Cell apoptosis.	Chronic venous disease (varicose veins, venous leg ulcer), vardiovascular (atherosclerosis, aneurysm), osteoarthritis, EMT, cancer development.
Stromelysins					
MMP-3	11q22.3	Endometrium, appendix, urinary bladder.	ECM, fibronectin, laminin, collagens III, IV, IX, and X, cartilage proteoglycans.	Tissue remodeling.	Fibrotic disorder (lung fibrosis, liver fibrosis), liver disease (cirrhosis, portal hypertension), osteoarthritis, cardiovascular (atherosclerosis, aneurysm) , cancer development.
MMP-10	11q22.2	Endometrium.	ECM, fibronectin, laminin, elastin, proteoglycan core protein, gelatins, and collagens III, IV.	Cell apoptosis, wound healing, tissue remodeling.	Chronic venous disease (varicose veins, venous leg ulcer), liver disease (cirrhosis,portal hypertension), lung disease (asthma, COPD), viral infection (adenovirus, influenza), cancer development.
MMP-11	22q11.23	Endometrium, placenta.	ECM.	Wound healing.	Cancer development.
Matrilysins					
MMP-7	11q22.2	Gall bladder, endometrium, urinary bladder, kidney, prostate.	ECM, proteoglycans, fibronectin, elastin and casein.	Cell apoptosis.	Lung disease (asthma, COPD),inflammation (increased cytokines), cardiovascular (atherosclerosis, aneurysm), cancer development.
MMP-26	11p15.4	endometrium	ECM, collagen type IV, fibrinogen, and beta-casein.	Tissue remodeling.	Chronic venous disease (varicose veins, venous leg ulcer), cancer development.
Membrane-type (MT) MMPs					
MMP-14	14q11.2	Endometrium, gall bladder, placenta, urinary bladder, appendix.	ECM.	Morphogenesis.	Cardiovascular (atherosclerosis, aneurysm), cancer development.
MMP-15	16q21	Thyroid, placenta,testis, colon, duodenum.	ECM.	Morphogenesis.	Cancer development.
MMP-16	8q21.3	Brain, gall bladder, endometrium, thyroid, ovary.	ECM.	Angiogenesis.	Cancer development.
MMP-17	12q24.33	Brain, skin, fat, spleen, ovary.	ECM.	–	Cancer development.
MMP-24	20q11.22	Kidney, brain, lung, prostate, fat.	ECM.	Immune response.	Neurological disease (neuropathic pain, decreased neural plasticity), cancer development.
MMP-25	16p13.3	Bone marrow, appendix, spleen, lung, lymph node.	ECM.	Cell apoptosis, Immune response.	Lung disease (asthma, COPD), cancer development.
Other MMPs					
MMP-12	11q22.2	Appendix, urinary bladder, colon, placenta, small intestine.	ECM.	Immune response.	Lung disease (asthma, COPD), cancer development, neurological disease (neuropathic pain, decreased neural plasticity), viral infection (adenovirus, influenza).
MMP-19	12q13.2	Gall bladder, appendix, spleen, placenta, lung.	ECM.	Angiogenesis, wound healing.	Osteoarthritis, neurological disease (neuropathic pain, decreased neural plasticity), lung disease (asthma, COPD), cancer development, liver disease (cirrhosis, portal hypertension).
MMP-20	11q22.3	Testis, appendix.	ECM, amelogenin.	Tooth enamel formation.	–
MMP21	10q26.2	Ovary, gall bladder.	ECM.	Embryogenesis.	Cancer development.
MMP-23	1p36.33	Placenta, prostate, gall bladder, lung, ovary.	ECM.	Reproduction menstruation, cell apoptosis.	Cancer development.
MMP-27	11q22.2	Skin, fat, bone marrow.	ECM.	Embryogenesis, reproduction menstruation.	Cancer development.
MMP-28	17q12	Fat, skin, testis, colon, lung.	ECM.	Tissue remodeling, embryogenesis.	Cardiovascular (atherosclerosis, aneurysm), cancer development.

chronic obstructive pulmonary disease (COPD); lung cancer (LC); extracellular matrix (ECM); epithelial-to-mesenchymal transition (EMT); matrix metalloproteinases (MMPs).

Lung cancer(LC) is the most frequently new diagnosed cancer, and it’s estimated that LC ranked the top one among cancer deaths in 2020 (6). High activity and overexpression of MMPs in LC tissues contributed to LC invasion and metastasis, which induced to poor survival outcomes in cancer patients (7, 8). Study showed that MMP loss induced cell apoptosis in non-small cell lung cancer (NSCLC) (9). In A549 and H1299 LC cells, high expression of MMP-9 and MMP-2 contributed to epithelial-to-mesenchymal transition (EMT) (10). Together, high expression of MMPs induced cancer cell stemness, replication, inflammation, et al (11–13). In this review, we focus on the multi-faced roles of MMPs in LC, and try to discover potential application of targeting MMPs in immunotherapy in LC patients.

## High MMPs are associated with cancer diagnosis, treatment and poor outcomes in patients with LC

**Figure 1:**
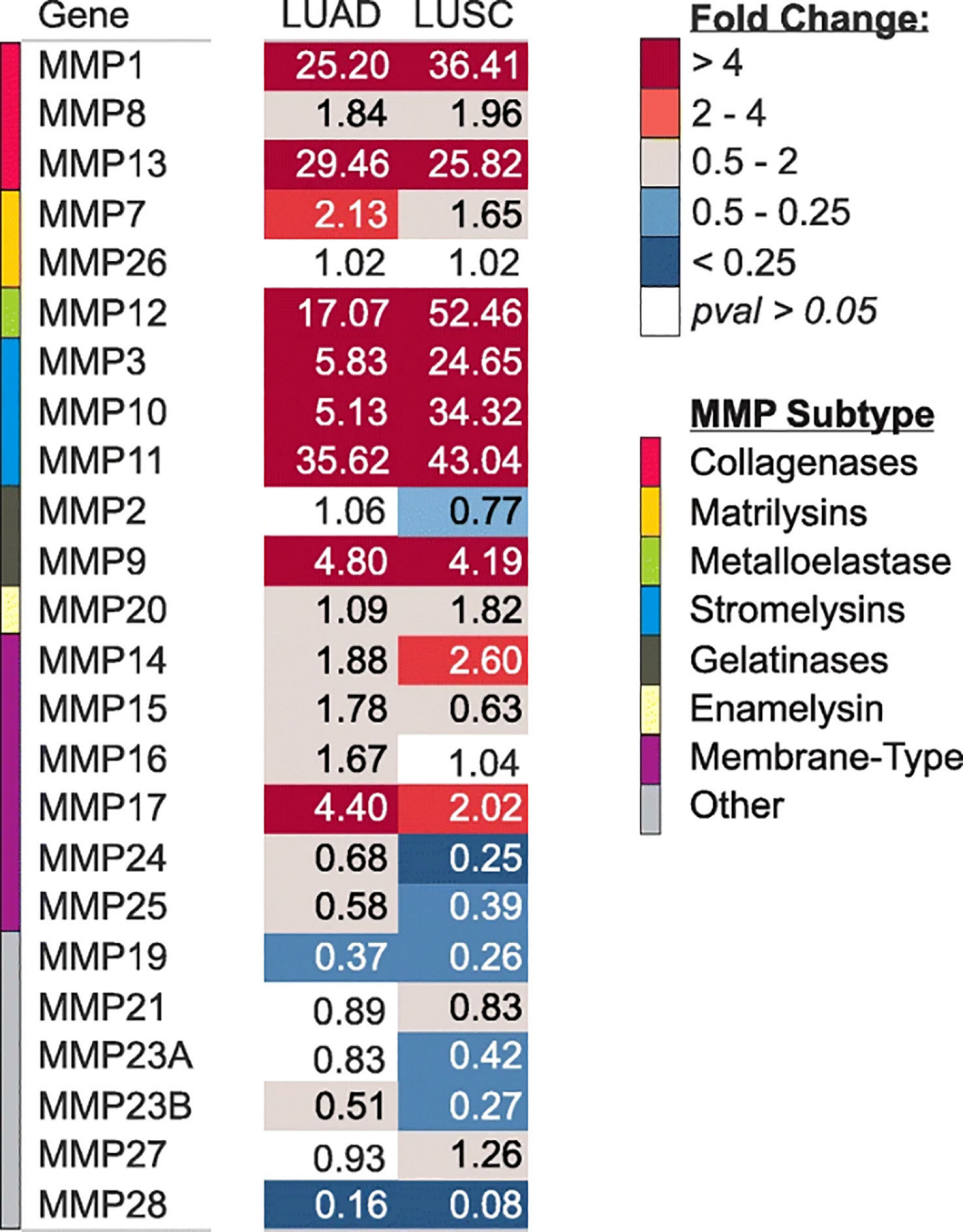
Gene expression of MMPs in LUAD and LUSC in TCGA. copyright 2009, with permission from Elsevier. Reprinted by permission from Springer Nature: Tumour Biol. Jiang J, Liu HL, Liu ZH, Tan SW, Wu (B) Identification of cystatin SN as a novel biomarker for pancreatic cancer. 2015;36 (5):3903–3910. Copyright 2015.

Expression of MMPs were significantly correlated with TNM stage and poor survival outcomes in LC patients (14). A study has clearly investigated MMPs expression in lung adenocarcinoma (LUAD) and lung squamous cell carcinomas (LUSC) tissues (**Figure 1**). The results showed that MMP-1, 3, 9, 10, 11, 12, 13, and 17 were significantly upregulated in both LUAD and LUSC tissues (**Figure 1**). It’s reported that high expression of MMP-1 was related to male gender, smoking, and poorly differentiated tumor, besides, high serum MMP-1 showed a trend for short overall survival (14, 15) (**Table 2**). In addition, high expression of MMP-1 was also associated with tumor initiation, invasion, and metastasis in LC (34). MMP-2 was highly expressed in LC tissues, and adenocarcinomas showed higher expression of MMP-2 compared with squamous cell or large cell carcinomas (16, 17). Univariate and multivariate analyses showed that expression of MMP-2 showed significant prognostic value and predicated tumor recurrence in patients with NSCLC (16). Study also showed that MMP-2 may be implicated in early-stage tumor invasion, metastasis, and angiogenesis in NSCLC (35). Interestingly, in another study, MMP-2 expression didn’t show significant variation between metastatic and non-metastatic LC patients (36). The expression levels of MMP-3 differed significantly between patients with low and high N stage (P<0.001) (14). Higher MMP-3 expression levels were correlated with higher stages, therefore, MMP-3 was a potential marker associated with a high overall stage (P<0.001) (14). Study showed that expression of MMP-7 and MMP-9 were significantly higher in tumor tissue than in the adjacent tissues (37). Overexpression of MMP-7 played a role in cancer metastasis, and was associated with poor prognosis in NSCLC, besides, MMP-7 was involved in physiological processes including pathogenesis, invasion, and metastasis, contributing to predict the progression and prognosis of NSCLC (38, 39). However, another study showed that MMP-7 and MMP-9 may not be markers in early-stage tumor invasion, metastasis, and angiogenesis in NSCLC (35). MMP-9 expression in tissue was significantly higher in NSCLC than in small cell lung cancer (SCLC), and MMP-9 overexpression in NSCLC was related to the pathologic type and clinical stage of NSCLC, indicating its potential as a therapeutic target (19). MMP-9 expression differed significantly between cases with low and high N stage (P=0.025), and it was also correlated with overall stage, though without statistical significance (14). In addition, *in vivo* experiments showed that MMP-9 expressions of stage III and stage IV LC tissues were significantly higher than that in stage I and stage II LC tissues, indicating MMP-9 might be used as therapy and prognostic indicators for LC (20). Shorter survival time was found among positive MMP-9 expression in stage I NSCLC patients with negative lymph node (40). Interestingly, in this study, no difference in overall survival was observed with MMP-9 expression (41). In NSCLC tissues, the MMP-10 mRNA level was positively correlated to the MMP-10 protein level, however, there is no correlation in the adjacent tissues (42). MMP-10 plays an important role in the recurrence of stage IB LC patients, no matter what the histologic type is (43). However, univariate analysis showed that MMP-10 expression has no statistical significance with patients’ survival (P<0.289) (14). MMP-11, the highest upregulated MMP family member in LUAD cells, was also significantly increased in LUAD tissues (21). Besides, MMP-11was found to be upregulated in the recurred stage IB LC (43). *In vitro* study showed that MMP-11 depletion severely impaired cell proliferation, migration, and invasion of A549 LUAD cell, indicating MMP-11 is a key cancer driver gene in LUAD and is a potential target for cancer therapy (21). Expression of MMP12 protein was significantly increased in LUAD tissues compared with adjacent normal tissues (p= 0.019), and was closely correlated with the pathological stage and lymph node metastasis of LUAD patients (p= 0.01; p= 0.003), therefore, MMP-12 may be a promising therapeutic target for LUAD patients (22, 23). Immunostaining analyses showed that high MMP-13 index was found in most of the invasive LUAD lesions (24/27), but in none of the non-invasive tissues(0/4) (p=0.001) (44). Besides, MMP-13 positive expression accounts for larger proportion in grade II LC patients than grade I (24). MMP-14 was found to be upregulated in the recurred stage IB LC (43). In addition, high expression of MMP-14 is an unfavorable prognostic factor, and high levels of MMP-14 protein were positively correlated with advanced clinical stage, higher N classification, distant metastasis, and lower differentiated degree in patients with NSCLC (25, 26). Study showed that MMP-15(MT2-MMP)was also upregulated in the recurred stage IB LC (43). Besides, both mRNA and protein expression levels of MMP-15 were significantly upregulated in NSCLC tissues compared with adjacent normal tissues, in addition, MMP-15 might play an important role in promoting the tumor progression and angiogenesis in NSCLC (27). MMP-19 gene and protein expression were increased in LC tumors compared with adjacent normal tissues. The results derived from three independent datasets showed that increased MMP-19 gene expression conferred a poorer prognosis in NSCLC. Besides, *in vitro* experiments showed that overexpression of MMP-19 promotes EMT, migration, and invasiveness in several NSCLC cell lines (45). Study showed that the expression of MMP-20 in tumor tissues is very restricted, while in this study, expression of MMP-20 in LC was clearly observed and extremely low MMP-20 expression in normal tissues (28). Like most of MMP family members, MMP-24(MT5-MMP) was also overexpressed in LC tissues (29). Similarly, MMP-26 expression in LC tissues were also significantly higher than that in para-cancerous tissues, besides, high MMP-26 protein level was correlated to carcinogenesis, grade of cell differentiation, TNM stages, lymph node metastasis, and prognosis of NSCLC. Therefore, MMP-26 might be a potential tumor marker in monitoring progression and predicting prognosis of NSCLC (30–32). Study showed that MMP-27, MMP-28 were not significantly upregulated in LUSC and LUAD (46), suggesting that minor role of MMP-27, 28 played in NSCLC. Together, high expression of MMPs in LC tissues was significantly related with poor survival outcomes, advanced stages, larger tumor sizes, et al, indicating MMPs might be potential targets for LC therapy and useful markers for LC detecting.

**Table 2:** Analyses of MMPs expression in lung cancer.

MMP	Case of patient	MMPs expression (IHC)	Differentiation/Distant metastasis	Stage	TNM	Survival	Reference
MMP-1	43	Upregulated in NSCLC.	–	–	–	Univariate analysis: OS: p=0.034; RFS: p=0.990. Multivariate analysis: OS: p=0.138.	(14)
	85	Upregulated in NSCLC.	Differentiation well to medium/low: p= 0.057.	Stage I and II/ III and IV: p=0.022.	N0-1/N2-3: p= 0.021.	–	(15)
MMP-2	212	Upregulated in NSCLC	Differentiation well to medium/low: p=0.036.	Stage I–II/III- IV: p=0.163.	T1–T2/T3: p=0.931; N0-1/N2-3: p=0.109.	Univariate analysis: OS: p=0.010; DFS: p=0.045. Multivariate analysis: OS: p=0.028; DFS: p=0.039.	(16)
	125	Upregulated in NSCLC.	–	Stage I–II/III: p=0.009.	T1–T2/T3: p=0.061; lymph node metastasis: No/Yes: p=0.027.	Median OS: 30.53 months [95% CI =26.69–33.31] vs. 40.06 months [95% CI=33.55–46.45], p=0.022.	(17)
MMP-3	43	Upregulated in LC.	–	Stage I and II / III and IV: p<0.001.	N0-1/N2-3: p<0.001.	Univariate analysis: OS: p=0.360.	(14)
	NSCLC: 1926; LUAD: 720; LUSC:524	Upregulated in NSCLC.	–	–	–	Univariate analysis: OS(NSCLC): HR=1.14[95%CI =1.002-1.30], p=0.042; OS(LUAD): HR=1.45[95%CI =1.14-1.84], p=0.0021; OS(LUSC): p=0.35.	(18)
MMP-7	90	Upregulated in NSCLC.	Differentiation well to medium/low: p=0.001.	–	lymph node metastasis: No/Yes: p=0.001.		(19)
MMP-8	–	–	–	–	–	–	–
MMP-9	43	Upregulated in NSCLC.	–	–	N0-1/N2-3: p=0.025.	Univariate analysis: OS: p=0.124; Multivariate analysis: OS: p=0.320.	(14)
	50	Upregulated in NSCLC.	Distant metastasis No/Yes: p= 0.0071.	Stage I-II/III-IV: p=0.012.	Lymph node metastasis: No/Yes: p=0.0326.	–	(20)
MMP-10	43	Upregulated in NSCLC.	–	–	–	Univariate analysis: OS: p=0.289.	(14)
	32	Upregulated in NSCLC.	–	Stage I/II-III: p=0.8050.	T1/T2: p=0.8780; lymph node metastasis No/Yes: p= 0.8050.	–	(21)
	20	Upregulated in NSCLC.	–	–	–	Univariate analysis: OS: p=0.0396.	(22)
MMP-11	20	Upregulated in LUAD.	–	–	–	–	(22)
	GEO and TCGA datasets	Upregulated in LUAD.	–	–	–	–	(23)
MMP-12	52	Upregulated in LUAD.	Differentiation well to medium/low: p=0.205;	Stage I-II/III-IV: p=0.01.	Tumor size (cm) ≧̸5/<5: p=0.587; lymph node metastasis No/Yes: p=0.003.	–	(24)
MMP-13	31	Upregulated in LC.	Invasive lesions /non-invasive lesions: p= 0.001.	–	–	–	(25)
	56	Upregulated in LC.	–	–	Lymph node metastasis No/Yes: p=0.438.	–	(26)
MMP-14	104	Upregulated in NSCLC.	Differentiation well to medium/low: p=0.001; distant metastasis No/Yes: p=0.014.	Stage I–II/III–IV: p<0.001.	N0–N1/N2–N3: p<0.001.	Univariate analysis: OS: p<0.001.	(27)
MMP-15	85	Upregulated in NSCLC.	Nadal metastasis No/Yes: p=0.0483.	Stage I/II-III: p=0.0483.	T1/T2: p=0.6606.		(28)
MMP-16	–	–	–	–	–	–	
MMP-17	–	–	–	–	–	–	
MMP-19	408(2 datasets)	Upregulated in NSCLC.	–	–	–	OS in the dataset 1 HR=1.59[95%CI=1.19–2.13]; p= 0.001. OS in the dataset 2 HR=2.28[95%CI=1.00–5.21]; p= 0.033.	(29)
MMP-20	0	Upregulated in LC cells(RT-PCR).	–	–	–	–	(30)
MMP-21	–	–	–	–	–	–	
MMP-23	–	–	–	–	–	–	
MMP-24	Unclear	Upregulated in LC.	–	–	–	–	(31)
MMP-25			–	–	–	–	
MMP-26	70	Upregulated in LC.	Differentiation well to medium/low: p<0.01.	Stage I-II/III-IV: p<0.01.	Tumor size (cm) <3/≥3: p>0.05.	–	(32)
MMP-27	TCGA	Not significantly upregulated in LUSC and LUAD.	–	–	–	–	(33)
MMP-28	TCGA	Not significantly upregulated in LUSC and LUAD.	–	–	–	–	(33)

lung cancer (LC); non-small cell lung cancer (NSCLC); lung adenocarcinoma (LUAD); lung squamous cell carcinomas (LUSC) ; matrix metalloproteinases (MMPs); recurrence-free survival (RFS); overall survival (OS); disease-free survival (DFS); confidence interval (CI); hazard ratio (HR).

## MMPs are involved in the resistance of LC to anti-tumor therapies

Accumulated evidence showed that MMPs were closely involved in anti-tumor treatment effects in patients with LC.

Overexpression of MMPs significantly contributed to EGFR-TKI-resistance in LC cells (**Table 3**). For example, upregulation of MMP-1, following the activation of mTOR signaling pathway, plays a significant role in migration and invasion of gefitinib- and erlotinib-resistant PC-9 cells, indicating that targeting MMP-1 and mTOR signaling pathways in EGFR-TKI-resistant LUAD might be potential therapy (47). In gefitinib-resistant NCI-H460 LC cells, MMP-2 is highly expressed, and MMP-2 suppression significantly inhibit cell adhesion, migration and invasion (33). In gefitinib-resistant NSCLC cells, MMP-12 was inhibited through suppression of the EGFR/ERK signaling pathway, thus inhibited cell proliferation, invasion, and migration (48). In sunitinib-resistant LC cells, upregulation of MMP-2/9 contributed to cell migration, invasion and metastasis (49). In the crizotinib-resistant A549 cells, increased expression of MMP-9 induced EMT, enhanced cell invasion and migration (50). Together, overexpression of MMP-1, 2, 9 induced EGFR-TKI-resistance in LC cells, inhibiting their expression might be useful to restore effects of EGFR-TKI on LC cells.

**Table 3:** MMPs are involved in the resistance of lung cancer to anti-tumor therapies.

Cell types	MMP expression	Molecules or signaling pathways involved	Biological process	Effects	Reference
EGFR-TKI-resistant LC cells					
gefitinib- and erlotinib-resistant PC-9 cells	upregulation of MMP-1	mTOR signaling	Cell migration and invasion	Positive	(47)
gefitinib-resistant NCI-H460 LC cells	upregulation of MMP-2	p-FAK, p-ERK1/2, Ras and E-cadherin.	cell adhesion, migration and invasion	Positive	(33)
gefitinib-resistant A549 and H1975 cells	upregulation of MMP-12	EGFR/ERK signaling	cell proliferation, invasion, and migration	Positive	(48)
sunitinib-resistant lewis cells	upregulation of MMP-2,9	TGFβ	cell migration, invasion and metastasis, EMT	Positive	(49)
crizotinib-resistant A549 cells	upregulation of MMP-9	CD74, ROS1	EMT, cell invasion and migration, decreased cell apoptosis	Positive	(50)
platinum resistance					
platinum-resistant HCC827 and A549 cells	upregulation of MMP-2,9	ROS-p53, AIF, PARP	Cell apoptosis	Negative	(51)
cisplatin-resistant A549 cells	upregulation of MMP-2,9	lncRNA ZXF1, MAPK pathway	Increased cisplatin resistance and cancer progression	Positive	(52)
cisplatin-resistant A549 cells	upregulation of MMP-9	E2F1, Rb	Cell cycle cell migration and invasion.	Positive	(53)
A549 cells	upregulation of MMP-9	caspase-3,9, P53, Akt/IKKa pathway	Cell proliferation, decreased apoptosis, cell invasion and migration, decreased carboplatin sensitivity	Positive	(54)
radiotherapy-resistant-					
radiotherapy-resistant NCI-H23 cells.	upregulation of MMP-2	caspase-3, caspase-3, Rad50.	Cell survival and decreased radiation sensitivity	Positive	(55)
radiation-resistant NCl-H1650 cells	upregulation of MMP-2,9	Sp1	cell migration and invasion, decreased radiation sensitivity	Positive	(56)
A549, H460, and H1299 cells	upregulation of MMP-2,9	Nrf2/Notch signaling	EMT, cell migration, decreased radiation sensitivity	Positive	(57)
A549 and H1299 cells	upregulation of MMP-2,9	cyclin D1	Cell proliferation, cell invasion, cell cycle, decreased radiation sensitivity	Positive	(58)
anti-angiogenetic drugs Patients with NSCLC	upregulation of MMP-9	SDF-1a, HIF-1a, bFGF	increased resistance to anti-angiogenetic drugs	Positive	(59)
cytotoxic drugs, A549 cells	upregulation of MMPs	TIMP-2	Decreased chemosensitivity	Positive	(60)

lung cancer (LC); non-small cell lung cancer (NSCLC); lung adenocarcinoma (LUAD); lung squamous cell carcinomas (LUSC) ; matrix metalloproteinases (MMPs); recurrence-free survival (RFS); overall survival (OS); disease-free survival (DFS); confidence interval (CI); hazard ratio (HR).

MMPs are greatly involved in platinum resistance in LC cells (**Table 3**). Study showed that MMP-2/9 expression are significantly increased in HCC827 and A549 platinum-resistant LC cells, inhibition of the MMP-2/9 signaling pathways significantly induces cell apoptosis in cisplatin-resistant LC cells (51). In cisplatin-resistant LC cells, lncRNA ZXF1 contributes to cisplatin resistance and leads to the poor prognosis of LC patients by activating MAPK pathway and MMP 2/9 (52). Similarly, tyrphostin AG-1478, a selective EGFR-TKI, provide a potential therapeutic approach for cisplatin-resistant LC patients by inhibiting MMP-9 expression (53). In NSCLC cells, Curcumin(derived from the plant Curcuma longa) combined with carboplatin significantly inhibited tumor growth, cell migration, and invasion by effectively inhibiting MMP-2/9 expression, indicating that targeting MMP-2/9 and decreasing their expression and activities were potential effective way to enhance carboplatin sensitivity in patients with NSCLC (54). Together, inhibition of MMP-2, 7, 9 might be useful for restoring sensitivity of LC cells to platinum.

MMPs expression also affected radiation sensitivity (**Table 3**). MMP-2 is highly expressed in radiotherapy-resistant or radiotherapy-insensitive LC cells. Suppression of MMP-2 level significantly reduced, inhibited survival of cancer cells, and promoted radiosensitization (55). Overexpression of pecificity protein 1 (Sp1) lead to upregulation of MMP-2/9 in radiation-resistant LC cells. Celecoxib significantly restored radiation sensitivity and inhibited cell migration and invasion by inhibiting the expression and activity of Sp1, and MMP-2/9 (56). It’s reported that downregulation of MMP2/9 by inhibition of nuclear factor E2 related factor 2 (Nrf2) significantly reduced EMT, thus increased radiosensitivity of NSCLC cells (57). Interestingly, the proteasome inhibitor MG132, could regulated cell cycle and MMP 2/9 expression, which enhanced sensitivity of NSCLC cells to radiotherapy (58). In conclusion, reduced expression of MMP-2, 9 significantly enhanced sensitivity of LC cells to radiotherapy.

MMPs also affected other anti-tumor therapies (**Table 3**). Pre-clinical studies have showed that overexpression of MMP-9 contributed to resistance to anti-angiogenetic drugs (59). In A549 LC cells, TIMP-2 impeded tumor progression by inhibiting MMPs expression, which increased chemosensitivity of A549 cells to cytotoxic drugs, indicating that manipulating MMPs expression are potential approach to restore sensitivity of LC cells to cytotoxic drugs (60).

All in all, MMPs expression are closely associated with resistance of LC to anti-tumor therapies, targeting MMPs might be useful alternative to restore sensitivity of LC to anti-tumor therapies.

## Regulatory network of MMPs in LC

**Figure 2:**
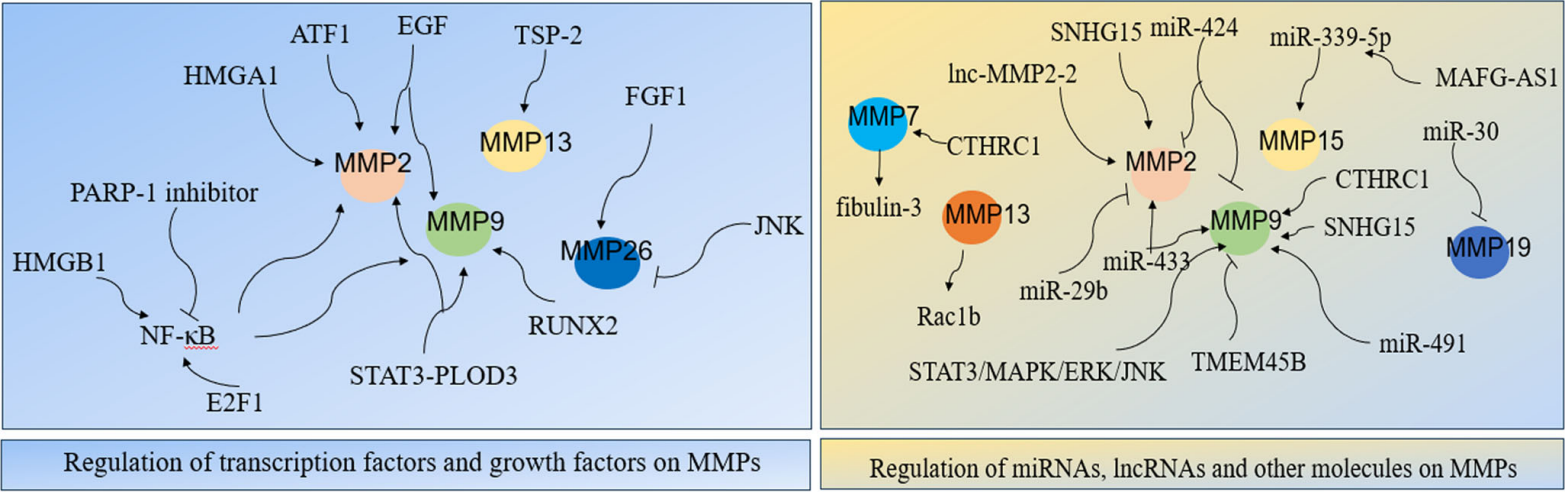
Regulatory network of MMPs in LC.

MMPs were widely involved in various signaling network that are important to LC cells. Studies showed that transcription factors, growth factors, miRNAs and lncRNAs were related with expression and regulation of MMPs, which significantly affected LC development (**Figure 2**).

Transcription factors nuclear transcription factor-κB(NF-κB), E2F1, HMGA1, RUNX2, STAT3, and activating transcription factor 1 (ATF1) were important for expression and regulation of MMPs. NF-κB was firstly discovered in 1986, it regulates expression of its target genes and it plays crucial roles in cancer initiation and progression (61, 62). Studies have shown its role in MMPs in LC cells. Specifically, NF-κB stimulates the secretion of MMP-2/9, thus induced LC metastasis (63). In NSCLC cells, carbon ion (12C) radiotherapy combined with PARP-1 inhibitor significantly inhibited NF-kB expression, which followed by reduced MMP-2/9 expressions and significantly suppressed EMT and cancer metastasis (64). Study showed that suppressing MMP-9-dependent invasion pathway by regulating NF-κB activity was useful to inhibit ionizing radiation-induced LC metastasis (63). In LC A549 cells, curcumin suppressing cell migration and invasion by inhibiting adiponectin through NF-κB/MMP pathways, indicating that targeting NF-κB/MMP pathways might be an alternative for adjuvant therapy in LC patients (65). In SCLC cells, MMPs transcription could be directly enhanced by transcriptional activator E2F1 or be indirectly activated through enhanced NF-κB as a consequence of E2F1 activation (66). In LC, overexpression of TSP-2 leads to activation of integrin αvβ3/FAK/Akt/NF-κB/MMP-13 signaling pathway, which hence enhanced cell migration and invasion (67). The nucleoprotein HMGB1, promoted LC invasion and metastasis by upregulating the expression and activity of MMP-2 in an NF-κB-dependent manner (68). The architectural transcription factor HMGA1, contributed to transformation in undifferentiated large-cell LC by upregulating MMP-2 (69). RUNX2 is a Runt-related transcription factor, and it was aberrantly activated in cancer progression (70). In LC H1299 cells, inhibition of RUNX2 and its target gene MMP-9 by WW domain-containing oxidoreductase (WWOX) significantly suppressed cancer cell migration and invasion (71). Transcription factor STAT3 interacts with PLOD3, which hence lead to MMP-2/9 expression and contributed to LC metastasis (72). In LC cells, upregulation of activating transcription factor 1 (ATF1) increased MMP-2 expression, thereby causing cell invasion and migration (8). Together, MMPs expression largely depends on the regulation of transcription factors.

Growth factors are important proteins that regulate cell growth and proliferation, and they exert their functions through binding with specific receptors (73). Increasing evidences indicated that growth factors and their receptors are involved in MMPs regulation in LC. In NSCLC cells, four growth factors: insulin-like growth factor I/II (IGF I/II), hepatocyte growth factor (HGF), and epidermal growth factor (EGF) contributed to cell migration and invasion by increasing the expression and activity of MMP-2/9 (74). In A549 LC cells, suppressing p-EGFR lead to downregulation of MMP-2/9 expression, which hence inhibited cell proliferation, migration and invasion (75). Interestingly, overexpression of Derlin-1 in NSCLC contributed to cell invasion through EGFR-ERK-mediated up-regulation of MMP-2/9 (76). In A549 LC cells, fibroblast growth factor 1 (FGF1) -induced p-FGFR1 activated MMP26, which hence lead to cancer invasion, besides, inhibition of JNK significantly decreased the activation of MMP26 in response to FGF1 stimulation (31). All in all, growth factors and their receptors are closely involved in MMPs expression and activation, targeting or inhibiting combination of growth factors and their receptors might be potential effective ways to suppress MMP-induced LC invasion and metastasis.

More and more evidences indicated that miRNA interferes with MMPs expression. MiR-29b negatively regulated MMP-2, which might have potential usefulness for the treatment of NSCLC (77). MiR-30 isoforms (a microRNA family targeting MMP-19) are significantly down-regulated in human LC and regulate MMP-19 expression (45). Study showed that regulation of miR-339-5p/MMP15 axis by lncRNA MAFG-AS1promoted cell metastasis in NSCLC (78). In A549 cells, miR-424 inhibited MMP-2/9 expression, which thereby decreasing cell proliferation and migration (79). It’s reported that upregulation of miR-433 is closely related to decreased expression of MMP-2/9, which hence inhibited cell proliferation and invasion in NSCLC (80). In A549 cells, ginsenoside Rh2 (G-Rh2), derived from ginseng, exerts its anti-metastasis activity by repressing MMP-9 expression through miR-491, thereby inhibiting cancer metastasis (81). All in all, miRNAs are important for regulation of MMPs expression in LC.

LncRNA, is also significant for MMPs regulation in LC. In NSCLC, lncRNA MAFG-AS1 promoted cell metastasis by regulating miR-339-5p/MMP15 signaling pathway (78). TGF-β-mediated lnc-MMP2-2 regulates migration and invasion of LC cells by increasing MMP-2 expression (82). In A549 cells, knocking down lncRNA SNHG15 inhibited cell invasion and metastasis by suppressing the MMP-2/9 expression and EMT (83). In conclusion, lncRNA is greatly involved in expression and activation of MMPs.

Other molecules were also significant for MMPs network. Study showed that MMP3-Rac1b signaling axis is the important driver of tumor progression in LC (84). Downregulation of fibulin-3 contributes to LC invasion and metastasis by increasing MMP-7 expression (85). In NSCLC, overexpression of CTHRC1 contributes to cancer invasion and metastasis in a MMP7- and MMP9-dependent manner (86). In LC cells, inhibition of transmembrane protein 45B (TMEM45B) significantly inhibited cell invasion by regulating the expression of MMP-9 (18). Study showed that down-regulation of phosphorylation of JAK2/STAT3 significantly inhibited its downstream target genes MMP-2/9, thereby suppressing cancer metastasis and invasion in NSCLC (87). ERK activation in LC resulted to MMP-9 expression, thus led to cancer migration and development (88). In A549 cells, overexpression of spleen tyrosine kinase (Syk) significantly inhibited invasive ability of LC by inhibiting MMP-9 expression (89). In Lewis cells, suppressing the activities of MMP-2/9 through the modulation of STAT3/MAPK/ERK/JNK signaling pathway significantly inhibited cancer metastasis (90).

In conclusion, regulation of MMPs is complicated, and the regulatory network mainly included transcription factors, growth factors and their receptors, miRNA and lncRNAs, indicating that targeting or manipulating those regulators might be potential effective ways for LC therapy.

## MMPs induced cancer stem cell-like cells

Cancer stem cells (CSC) are important factor of tumor recurrence, harboring CSC-like properties like self-renewal, aberrant differentiation, tumor recurrence, and acquired therapeutic resistance (91). Studies have shown that MMPs induced CSC-like cells in LC. Lung cancer stem-like cells (LCSLCs) from LUAD A549 cells carry the self-renewal potential, increased invasion, elevated tumorigenic activity, and high expression of stemness markers CD133, CD44, and aldehyde dehydrogenase 1 (ALDH1). Anti-tumor treatment suppressed properties of LCSLCs, inhibited expression of stemness markers, and also reduced MMP-9 activity (92). The CD133+ lung cancer stem cell (LCSC) is significantly correlated to the tumor metastasis and patients’ survival. Upregulation of MMP-9 induced by CD133+ LCSC contributed to cancer invasion and metastasis[96]. In NSCLC, cancer recurrence and metastasis are closely related to CSC, and these cells induced MMP-9 secretion (93). In LC cells, Aiolos overexpression promotes CSC-like properties by upregulating MMP-16 (94). Study showed that in the co-culture system of LUAD A549 cells, esophageal cancer cells, and mesenchymal stem cells (MSCs)-conditioned medium, MSCs induced cell apoptosis and downregulated MMP-2 *in vitro*, interestingly, this study also showed that MSCs enhanced tumor formation and growth *in vivo (* 95). This result is really confusing, more vivo and vitro need to be done to figure out the phenomenon. In another study, cell fusion between LC cells and MSCs exerted increased metastatic capacity with upregulation of MMP-2/9 (96). Highly induced MMP-10 in lung bronchioalveolar stem cells (LBASCs) activated Kras, thus further contributed to tumor initiation, and maintained stem-like features (97). In conclusion, MMPs might be useful as diagnostic and prognostic biomarkers in LC, and may also represent a novel therapeutic approach to target LCSC.

## MMPs are involved in tumor immunity

Studies showed that MMPs are involved in tumor immunity by regulating innate immunity through affecting proinflammatory cytokines, chemokines, other immune-related proteins, tumor microenvironment (TME) and several kinds of innate immune cells (98). Inhibition of MMPs has been showed to be effective in stimulation of immune system and inhibition of tumor growth (99).

Studies showed that MMPs functions in the tumor microenvironment (TME), and also affect immunotherapy effects. Accumulated evidence showed that MMPs promotes cancer immunosuppression, angiogenesis and inflammation in TME (100). MMP-9 is important in promoting the extravasation of tumor cells in TME (41). Myeloid-derived suppressor cells (MDSCs) are major components of the immune suppressive TME. MDSCs enhanced the pro-angiogenic, immune suppressive and pro-tumorigenic behavior of cancer cells by upregulating MMP-9 (101). Correspondingly, inhibition of MMP-9 by monoclonal antibody and TIMP-1 decreased MDSCs, which may inhibit tumor’s evasion of the immune response (102).

Complicated relationship existed between MMPs and macrophages(Mφ). Mφ are major sources of MMP-1, infection and inflammation increased MMP-1 secretion in lung tissues, indicating MMP-1 is an important driver for lung immunopathology (103). In co-cultured LC cells, G-Rh2 significantly inhibited MMP-2/9 expression, thereby converting tumor-associated macrophages (TAMs) from M2 to M1 and inhibited cancer migration (104). TAMs induced the remodeling of ECM through MMP-2/9 release, and contributed to angiogenesis and lymphangiogenesis through MMP-9 release (105). In NSCLC, TAM expressed high levels of MMP-9, which significantly increased cell migration and invasion, participated in vessel formation and sprouting, and thus lead to cancer progression (106, 107). Interestingly, *in vivo* experiments showed that MMP-9 lead to anti-tumor immune response by inducing neutrophil infiltration. Surprisingly, MMP-9 activated tumor-infiltrating macrophages into a tumor-inhibiting phenotype, inhibited tumor growth and angiogenesis, indicating MMP-9’s potential in regulating the innate immune response into anti-tumor action (108). TAMs were reported to be extremely important in delivering pro-MMP-9, thus induced angiogenesis in TME (109). Interestingly, MMP-9 in turn enhanced recruitment of Mφ during infection (110). During cancer metastasis, C-X-C chemokine receptor type 7 (CXCR7) recruits tumor-promoting macrophages (M2) to tumor tissues and upregulated MMP-2/9 expression (111).

Neutrophils played an important role in tumor immunity involving MMPs. TANs have been shown to both promote and inhibit tumor development. TANs promoted tumor by contributing to degradation of ECM *via* NE and MMP-9 (112). MMP-8, the neutrophil collagenase, is an important regulator of innate immunity that has onco-suppressive functions in cancers, and inhibition of MMP-8 might exert complicated effects on innate immunity (113). Neutrophil-derived MMP-8 is increased during pulmonary infection, indicating crucial role of MMP-8 played in the immunopathology of infectious disease (114). Similar to TAMs, tumor-associated neutrophils (TANs) are critically important for proMMP-9 delivering to TME, which mediating angiogenesis in tumor tissues (109). Interestingly, MMP-9 also plays a crucial role in the transmigration of neutrophils, lymphocytes, and eosinophils. MMP-9 inhibition suppressed inflammatory cell migration by suppressing IL-1beta, IL-4, and TNF-alpha expression (115). Study showed that TANs are major source of MMP-9 in human head and neck cancer and hepatocellular carcinoma, and neutrophil-derived MMP-9 has been involved in the angiogenic switch and tumor growth (116). It is worth noting that neutrophil-derived MMP-9 is secreted in a TIMP1-free manner, thus harboring powerful ability of angiogenesis (117).

T cells were also involved in MMP-related tumor immunity significantly. Membranes of activated T cells strongly induced the production of MMP-1/9 in lung tissue macrophages (LTM), however, unstimulated T cells failed to induce the secretion of MMPs. Meanwhile, IL-1 and TNFα are also closely involved in this process (118), indicating important roles of proinflammatory cytokines played on MMP-1/9 release. *In vivo* experiment showed that depletion of CD4(+) T lymphocytes lead to inhibition of antitumor activity and decreased antibodies against MMP-2, indicating that contribution of immune response against MMP-2 might be potential novel tumor methods for cancer therapy (119). Interestingly, Anti-MMP-9 treatment increases expression of T cell-stimulating factors, such as IL-12p70 and IL-18 (120). *In vivo* experiments showed that combination of anti-MMP-9 and anti-PDL1 induced TCR diversity, increased CD3+ T cells(including memory/effector CD4 and CD8 T cells), suggesting that inhibition of MMP-9 increased T-helper cell 1 type cytokines, induced delivery of effector/memory T cells to tumor tissues (120).

MMPs expression affected function of natural killer (NK) cells. In NCI−H23 human NSCLC cells, combination of ionizing radiation and MMP-2 inhibition promoted the killing function of NK−92 natural killer cells to cancer cells, suggesting that MMP-2 suppression is helpful for restoring host anti-tumor immune response (121). Interestingly, in cancer cells, IL-8/17 induced enhanced activity of MMP-2/9, thus promoted cancer metastasis (122). It has been investigated that inhibition of MMP25 by siRNA enhances the ADCC capacity of NK cells, emphasizing the important functional role of MMP25 in the regulation of ADCC activity, indicating that inhibition of MMP25 activity could improve ADCC efficacy of therapeutically administered NK cells (123). Interestingly, *in vivo* experiments showed that MMP-25-null mice exhibit a defective innate immune response, suggesting the possibility of activating innate immunity through induction of the activity of MMP-25 for the treatment of immune-related diseases (98).

In conclusion, MMPs are involved in tumor immunity by mutually action with various innate immune components, indicating modulating MMPs regulation and secretion might be potential for activating innate immune response in cancer therapy.

## Effects of MMP polymorphisms on risk of LC

Polymorphisms in MMP genes were functional and may contribute to genetic susceptibility to cancers (124, 125).

Polymorphisms in the promoter region of MMP-1 significantly influenced the transcriptional activation of MMP1, as well as MMP-1 activity (126). A meta-analysis among diverse populations showed that MMP1-1607 2G/2G showed higher risk of LC than MMP1-1607 1G carriers (MMP1-1607 1G/1G and MMP1-1607 1G/2G), besides, MMP1-1607 2G carriers (MMP1-1607 2G/2G and MMP1-1607 1G/2G) showed significant higher risk of LC than MMP1-1607 1G/1G (34) (**Table 4**). In addition, a study showed that MMP1-1607 1G/2G showed higher risk of LC than MMP1-1607 1G/1G in never-smokers with odds ratio (OR) of 1.67 [95% confidence interval(CI)=1.02-2.76] (144), indicating 2G allele might be risk allele. However, a meta-analysis including 825 Han Chinese patients with LC showed no significant association between MMP1-1607 2G carriers and MMP1-1607 1G/1G, besides, another study showed that MMP1-1607 1G/2G and 2G/2G genotype (2G carriers) presented a lack of association with LC risk (OR = 1.04[95% CI = 0.68–1.58]and OR = 0.99[95% CI = 0.71–1.40], respectively), all of these results are consistent with the conclusion concluded by Xiao, X.Y etc. (34, 127, 139, 145)(**Table 4**). In addition, subgroup analyses by ethnicity identified significant association with LC risk in Asians, while ethnicity among Caucasian had no relationship with LC susceptibility (34) (**Table 4**), the results are consistent with the conclusion in another meta-analysis conducting on 24 studies with 10,099 LC cases and 9,395 controls (136). Possibly, genotypes varied between ethnic populations, therefore, there are differences of susceptibility to LC among various between ethnic populations. MMP1 2G/2G genotype enhances LC susceptibility especially in current and heavy smokers with OR=1.76 [95% CI= 1.26–2.39] and OR=2.55[95% CI=1.61–4.03], respectively, indicating prolonged cigarette exposure induced polymorphisms in MMP-1 (146) (**Table 4**). We speculated that smoking induced changes in tissues, which may build a microenvironment that is more suitable for tumorigenesis. Besides, microenvironment combined with increased MMP1 transcription might be important reason for that 2G/2G genotype have a higher risk of developing LC. A study showed that MMP1-1607 1G/2G polymorphism increased LC risk in Asians (131). While in another study, MMP-1 polymorphisms were found not associated with LC risk (135, 147). These findings are rather puzzling and contradictory, and we think they need to be further investigated in larger size of case–control studies. Based on the existing research results, we can speculate that Asians, especially Han Chinese, smoking and 2G carriers promote the risk of LC.

**Table 4:** Analysis of MMPs polymorphism in lung cancer.

Polymorphism	Reference	Article type, studies included/assay methods	Ethnicity: Cases/Controls	Risk of LC: OR [95% CI]; P-value	Results	Conclusion
MMP-1 (-1607 1G/2G)	(34)	Meta-analysis, 14 studies	Caucasian+Asian: 6068/5860	Total: 2G/2G vs. 1G/1G: 1.34 [1.09, 1.64]; 0.01. 2G/2G vs. 1G/1G+1G/2G: 1.26 [1.06, 1.49]; 0.01. 2G/2G+1G/2G vs. 1G/1G: 1.21 [1.05, 1.40]; 0.01. Caucasian: 2G/2G vs. 1G/1G:1.14 [0.90, 1.44]; 0.28. 2G/2G vs. 1G/1G+1G/2G: 1.18 [0.91, 1.54]; 0.21. 2G/2G+1G/2G vs. 1G/1G: 1.07 [0.95, 1.20];0.25. Asian: 2G/2G vs. 1G/1G: 1.61[1.16, 2.25]; <0.01. 2G/2G vs. 1G/1G+1G/2G: 1.32 [1.07, 1.64]; 0.01. 2G/2G+1G/2G vs. 1G/1G: 1.46 [1.11, 1.94]; <0.01.	2G allele might be risk allele; significant association with LC risk in Asians, while no relationship with LC susceptibility in Caucasian.	2G allele, Asians, especially Han Chinese, and smoking increased risk of LC. Ethnicity is an important factor affecting LC susceptibility. The results of these studies are conflicting, and need to be further investigated in larger size of case–control studies.
1G/1G			1150/1223			
1G/2G			2755/2722			
2G/2G			2163/1915			
					
MMP-1 (-1607 1G/2G)	(127)	Meta-analysis, -	Han Chinese.:825/825	2G/2G vs 1G/1G: 1.39[1.01-1.91];0.01. 2G/2G+1G/2G vs 1G/1G: 1.15 [1.02-1.30];0.207. 2G/2G vs 1G/1G+1G/2G: 1.25 [0.92-1.68];0.0001.	2G allele might be risk allele; no significant association between MMP1-1607 2G carriers and MMP1-1607 1G/1G; significant higher risk of LC in 2G/2G than 1G carriers.	
1G/1G			74 (9) /100(12.1)			
1G/2G			323 (39.2) /367 (44.5)			
2G/2G			428 (51.9) / 358 (43.4)			
					
MMP-1(-1607 1G/2G)	(128)	A case-control study, PCR-RFLP	Caucasian: 456/451	2G/2G vs. 1G/1G+1G/2G: 1.18 [0.91, 1.54]; 0.21. current smokers: 1.76 [1.29–2.39]; <0.05. heavy smokers: 2.55[1.61–4.03]; <0.05.	MMP1-1607 1G/1G was not associated with LC susceptibility in Caucasian; prolonged cigarette exposure increased risk of LC	
1G/1G			94 (20.62)/ 111 (24.61)			
1G/2G			152 (33.33)/ 196 (43.46)			
2G/2G			210 (46.05)/ 144 (31.93)			
MMP‐2 (‐1306 C/T)	(129)	A case-control study, PCR-DHPLC	Chinese, 781/852	CC genotype: nonsmokers: 2.38 [1.64–3.45]. light smokers: 5.55 [3.34–9.22]. heavy smokers: 10.25 [5.80–18.09]. smokers: CC vs CT or TT: 7.64[4.74–12.33] vs 4.26[2.57–8.44]; 0.0001. CC vs CT+TT: 2.18 [1.70–2.79].	C allele might be risk allele; MMP2 -1306C/T polymorphism was associated with risk of developing LC; smoking is an important risk factor of LC.	C allele and smoking might be risk factors. Ethnicity is an important factor affecting LC susceptibility. The results of these studies are conflicting, and need to be further investigated in larger size of case–control studies.
CC			644 (82.5)/585(68.7)			
CT			127 (16.3)/248 (29.1)			
TT			10 (1.2)/19 (2.2)			
					
MMP‐2 (‐1306 C/T)	(130)	Meta-analysis,7 studies	Caucasian+Asian: 3189/3013	Total: C vs. T: 1.1[0.93-1.44]; 0.19. CC vs. TT: 1.44[1.03-2.02]; 0.03. CC vs. CT/TT: 1.17[0.83-1.64]; 0.34. CC/CT vs. TT: 1.33[0.96-1.85]; 0.1. Caucasians: C vs. T: 0.96[0.78-1.17]; 0.66. CC vs. TT: 1.72[0.89-3.31]; 0.1. CC vs. CT/TT: 0.8[0.70-1.10]; 0.26. CC/CT vs. TT: 1.80[0.94-3.46]; 0.08. Asians: C vs. T: 1.32[1.15-1.52]. CC vs. TT: 1.36[0.91-2.01]; 0.13. CC vs. CT/TT: 1.39[1.03-1.87]; 0.03. CC/CT vs. TT: 1.19[0.81-1.75]; 0.39.	C allele might be risk allele in Asians but not in Caucasian.	
CC						
CT						
TT						
					
MMP‐2 (‐1306 C/T)	(131)	Meta-analysis,17 studies	Caucasian+Asian: 7983/ 7382	Total: 0.63 [0.46, 0.88]; 0.006. Caucasian:1.00 [0.54, 1.86]; 1.00. Asian: 0.58 [0.42, 0.81]; 0.001.	MMP2-1306 C/T polymorphism decreased LC risk in Asians.	
MMP2 −(‐1306 C/T)	(132)	Meta-analysis, 50 studies	Asian +European :1804/1867	0.50[0.43–0.59]; <0.05	MMP2 -1306 C/T was associated with lower susceptibility to LC.	
MMP2 -(‐735 C/T)	(130)	Meta-analysis,7 studies	Caucasian+Asian: 3189/3013	Total: C vs. T: 1.51[1.13-2.02]; 0.005. CC vs. TT: 1.88[1.17-3.02]; 0.009. CC vs. CT/TT: 1.60[1.14-2.24]; 0.006. CC/CT vs. TT: 1.63[1.01-2.62]; 0.04. Caucasians: C vs. T: 0.94[0.67-1.31]; 0.71. CC vs. TT: 1.03[0.29-3.70]; 0.96. CC vs. CT/TT: 0.91[0.62-1.35]; 0.65. CC/CT vs. TT: 1.07[0.30-3.82]; 0.91. Asians: C vs. T: 1.93[1.52-2.23]; -. CC vs. TT: 2.07[1.24-3.47; 0.006. CC vs. CT/TT: 2.14[1.82-2.51; -.	C allele might be risk allele; MMP2-735 C/C increased LC risk in Asian but not in Caucasians compared with MMP2-735 T/T.	C allele might be risk allele. Ethnicity is an important factor affecting LC susceptibility. The results of these studies are conflicting, and need to be further investigated in larger size of case–control studies.
CC						
CT						
TT						
					
MMP2 -(‐735 C/T)	(132)	Meta-analysis, 50 studies	Asian +European :859/867	0.65 [0.53–0.79]; <0.05	MMP2-735 C/T was associated with lower risk in LC.	
					
MMP2 -(‐735 C/T)	(133)	A case-control study, PCR-RFLP	-: 816/610		no association was found statistically significant with risk of developing LC for the MMP2 -735 polymorphisms.	
CC			596 (73.0)/ 465 (76.2)	reference		
CT			206 (25.2)/ 125 (20.5)	1.18 [0.83- 1.67]; 0.359.		
TT			14 (1.7) /20 (3.3)	0.86 [0.35- 2.13]; 0.749.		
MMP-3 (-1171 5A/6A)	(134)	A case–control study, 5’ nuclease assay (TaqMan)	Caucasian: 2014/1323	6A/6A vs 6A/5A: 1.76[1.04–2.97]; <0.05	MMP-3 -1171 6A/6A was associated with higher risk of LC compared with MMP-3 -1171 5A/6A.	MMP 3 polymorphisms were not associated with LC risk. MMP-3 -1171 6A/6A harbor higher risk of LC compared with MMP-3 -1171 5A/6A.
6A/6A			517 (26.0)/ 350 (26.0)			
6A/5A			1012 (50.0)/ 648 (50.0)			
5A/5A			485 (24.0)/ 325 (24.0)			
					
MMP-3(-1171 5A/6A)	(133)	A case-control study, PCR-RFLP	-: 716 /534		MMP3 -1171 5A/6A polymorphisms were not significantly associated with risk of developing LC.	
6A/6A			185 (25.8) /139 (26.0)	reference		
6A/5A			367 (51.3)/ 276 (51.7)	1.19 [0.83- 1.72]; 0.339		
5A/5A			164 (22.9) /119 (22.3)	1.17 [0.76- 1.81];0.483		
					
MMP-3 (-1171 5A/6A)	(135)	Meta-analysis, 3 studies	-: 2430 /2023	1.03 [0.89–1.20]; 0.66	MMP 3 polymorphisms were not associated with LC risk.	
						
MMP-7 (-181 A/G)	(136)	Meta-analysis, 24 studies	Asian: 635 /649	1.89[1.41–2.54]; <0.001	MMP7 -181 A/G were risk factors for LC.	G allele might be risk allele. The results of these studies are conflicting, and need to be further investigated in larger size of case–control studies.
MMP-7 (-181 A/G)	(137)	A case–control study, PCR–RFLP	-: 243/350	A/G + G/G vs A/A: 2.00 [1.23-3.24]; 0.004	G allele might be risk allele.	
MMP-7 (-181 A/G)	(138)	A case–control study, PCR-RFLP	-: 132/80	A/G: 3.471[0.430-27.984]; 0.243. A/A: 4.213[0.531-33.461];0.174.	MMP-7 -181 A/G polymorphisms were not significantly associated with LC susceptibility.	
MMP-8 (-17 C/G)	(139)	A case–control study, PCR-RFLP	-: 501/510	0.65[0.45–0.93]; 0.019	MMP-8 -17 C/G polymorphism was associated with significant decreased risk of LC.	MMP-8 -17 C/G polymorphism was associated with decreased risk of LC.
MMP-8(-799 C/T)	(140)	A case–control study, PCR	Taiwanese: 528/1081		MMP-8 -799 C/T polymorphisms may not be associated with susceptibility to LC in Taiwanese.	MMP-8 -799 C/T polymorphisms may not be associated with susceptibility to LC.
CC			188 (52.5) /351 (49.0)	reference		
CT			130 (36.3) /273 (38.1)	0.87 [0.68-1.23]; 0.3999		
TT			40 (11.2) /92 (12.9)	0.79 [0.51-1.24]; 0.3198		
CT+TT			170 (47.5) /365 (51.0)	0.83 [0.59-1.17]; 0.2807		
						
MMP-9(-1562 C/T)	(133)	A case-control study, PCR-RFLP	Caucasian: 879/803		MMP9 -1562 T/T genotype was associated with decreased risk of LC.	T allele might be protective factor. Ethnicity is an important factor affecting LC susceptibility. The results of these studies are conflicting, and need to be further investigated in larger size of case–control studies.
C/C			581 (76.2) /483 (74.4)	reference		
C/T			174 (22.8) /148 (22.8)	0.96[ 0.69- 1.35]; 0.830		
T/T			7 (0.9) /18 (2.8)	0.23 [0.06- 0.85]; 0.027		
					
MMP-9(-1562 C/T)	(141)	Meta-analysis, 4 studies	Asian+ Caucasian: 1202/1039	Total: 1.07[0.59-1.95]; <0.001. Caucasian: 0.84[0.68-1.03]; 0.600. Asian: 2.73[1.74-4.27]; <0.05.	MMP9 -1562 C/T polymorphisms were risk factors for LC among Asians but not among Caucasians.	
					
MMP-9(-1562 C/T)	(135)	Meta-analysis, 2 studies	-: 333 /440	1.03 [0.73–1.45]; 0.86	No association was found between MMP9 -1562 C/T polymorphisms and risk of LC.	
MMP-9(-1562 C/T)	(136)	Meta-analysis: 4 studies	Caucasian+ Asian:1202/1039	0.80 [0.67–0.97]; <0.05	MMP9 -1562 C/T genotype was associated with decreased risk of LC.	
MMP-9(-1562 C/T)	(131)	Meta-analysis: 4 studies	Caucasian+ Asian:1295/1189	Total: TT vs. CT/CC: 0.38[ 0.19–0.75]; 0.005. Caucasian: 0.27 [0.12, 0.62]; 0.002. Asian: 1.21[0.06, 25.94]; 0.90.	T allele might be protective factor; MMP9-1562 C/T polymorphism decreased LC risk in Caucasians but not in Asians.	
MMP-11(rs738791)	(142)	A case-control study, PCR-RFLP	Taiwanese: 358/716	TT vs CC: 1.30[0.82- 2.08]; 0.3905. CT vs CC: 1.17[0.83-1.53]; 0.3818.	Not associated with LC risk.	MMP-11(rs738791) was not associated with LC risk. More analyses of case-control studies need to be done.
						
MMP-12 (-1082A/G)	(134)	A case-control study, 5’ nuclease assay (TaqMan)	Caucasian: 2014/1323; 0.42	*Men: A/G + G/G* versus *A/A:* 1.51[1.04–2.09]; <0.05. Women: not associated.	the G allele might be risk factor in men.	the G allele might be risk factor in men. Gender might be an important factor affecting LC susceptibility.
A/A			1767 (88.0)/ 1180 (89.0)			
A/G			235 (12.0)/ 137 (10.0)			
G/G			12 (1.0)/ 6 (1.0)			
MMP-12 (-82A/G)	(134)	A case-control study, 5’ nuclease assay (TaqMan)	Caucasian: 2014/1323	not associated.	No association was found between the MMP-12 -82A/G polymorphism and risk of LC.	No association between the MMP-12 -82A/G polymorphism and risk of LC. More analyses of case-control studies need to be done.
A/A			1535 (76%)/ 1008 (76%)			
A/G			449 (22%)/ 289 (22%)			
G/G			30 (2%)/ 26 (2%)			
MMP-13 (-77 A/G)	(139)	A case-control study, PCR-RFLP	-: 501/510		MMP13 -77A/G polymorphism was not associated with LC risk.	MMP13 -77A/G might to some extent be protective factor. More analyses of case-control studies need to be done.
A/A			248 (49.5)/ 267 (52.4)	Reference		
A/G			208 (41.5)/ 197 (39.4)	1.24 [0.91–1.69]; 0.174		
G/G			45 (9.0)/ 42 (8.2)	1.23 [0.72–2.11]; 0.452		
					
MMP-13 (-77 A/G)	(143)	A case-control study, PCR-RFLP	Han Chinese: 245/258		AA genotype was associated with a decreased risk of NSCLC compared with the GG genotype.	
GG/ GG			43 (17.55) 31 (12.02)	reference		
AG/ GG			94 (38.37) 87 (33.72)	0.78 [0.45–1.35]; 0.37		
AA/ AA			108 (44.08) 140 (54.26)	0.56 [0.33–0.94]; 0.03		
					
MMP-13 (-77 A/G)	(136)	Meta-analysis: 3 studies	Caucasian+ Asian:1221 /1225	Asian :0.67 [0.47–0.96]	MMP13 -77A/G might be protective factors among Asian.	

lung cancer(LC); matrix metalloproteinases (MMPs); polymerase chain reaction(PCR); polymerase chain reaction-denaturing high-performance liquid chromatography (PCR-DHPLC); polymerase chain reaction–restriction fragment length polymorphism (PCR–RFLP).

It’s reported that MMP2 735C/T and 1306C/T polymorphisms are both associated with LC risk (128). The MMP2 -1306C/T polymorphism was significantly associated with risk of developing LC, and smoking is important risk factor of LC, indicating that MMP2 polymorphisms plays an important role in human carcinogenesis under smoking status. Interestingly, MMP2 -1306C/C polymorphisms showed higher risk than MMP2 -1306C/T and MMP2 -1306T/T, indicating that C allele might be risk allele in Han Chinese (129) (**Table 4**). Interestingly, a study showed that MMP2 -1306C/C and MMP-2 735C/C polymorphisms were significantly associated with higher LC risk in Asians but not in Caucasians compared with CT/TT, suggesting C allele’s risk feature varies among different ethnic population (130) (**Table 4**). However, it was found that MMP2-1306 C/T polymorphism decreased LC risk in Asians compared with patients in Caucasian (131) (**Table 4**). It is worth noting that a meta-analysis with more than 40,000 subjects showed that MMP2 -1306 C/T was associated with lower susceptibility to LC with OR= 0.50[95%CI=0.43-0.59], MMP2-735 C/T was associated with lower risk in LC (OR = 0.65[95%CI=0.53-0.79]) (132) (**Table 4**). Another meta-analysis even suggested that MMP2 -1306 C/T, MMP2 -735 C/T might be protective factors for LC (136). The results in this study also showed that MMP-2 1306C/T and -735C/T were significantly associated with protection against LC, with OR=0.53[95% CI=0.40-0.72] and OR=0.65[95% CI=0.53-0.79], respectively (135). Interestingly, in this case-control study, no association was found statistically significant with risk of developing LC for the MMP2 -735 polymorphisms (133) (**Table 4**).

MMP-2 gene expression was lower in homozygous -735CC LC patients than in those with CT or TT genotypes. Interestingly, the survival time was longer in patients with the MMP-2 -735T allele than in those with the CC genotype (148). The results are confusing because higher expression of MMP-2 usually indicates poor survival outcomes, therefore, the results showed be further investigated. A large cohort patients analysis showed that MMP-2-1306 CT/TT and CT genotypes showed significantly poor progression-free survival (PFS) in Caucasian patients with NSCLC, indicating MMP-2 polymorphisms might be potential prognostic markers in NSCLC (149). In addition, studies showed that MMP2 -735 T/T genotype was also proved a statistically significant independent prognosis factor associated with poor survival in NSCLC patients(hazard ratio (HR) = 1.79[95% CI=1.00-3.20]) (133, 141). However, a study showed that MMP-2 -1306C/T has no significant association with survival in stage I NSCLC patients and was not associated with risk of LC (145, 147). All in all, C allele and smoking might be risk factors of LC. Besides, ethnicity is also an important factor affecting LC susceptibility. Importantly, the results of these studies are conflicting, and need to be further investigated in larger size of case–control studies.

A study including 2014 Caucasian LC patients and 1323 healthy controls showed that MMP-3 -1171 6A/6A was associated with higher risk of LC in never smokers compared with MMP-3 -1171 5A/6A (OR=1.76[95%CI=1.04–2.97]) (134) (**Table 4**). However, in another study, MMP 3 polymorphisms were not associated with LC risk (135) (**Table 4**), and MMP3 -1171 5A/6A polymorphisms were also not significantly associated with risk of developing LC (133) (**Table 4**). A case-control study including 382 patients with stage I NSCLC showed that MMP-3 6A/5A was not significantly associated with recurrence-free survival(RFS) and overall survival(OS) (145). It’s evaluated that MMP3 -1171 5A/6A was also not associated with survival of LC patients (133). Together, MMP 3 polymorphisms were not associated with LC risk, and MMP-3 -1171 6A/6A harbor higher risk of LC compared with MMP-3 -1171 5A/6A.

A meta-analysis suggested that and MMP7 -181 A/G were risk factors for LC among Asian (136) (**Table 4**). A study including 243 NSCLC patients and 350 healthy controls showed that the frequency of the MMP-7 -181G allele in NSCLC patients was significantly higher than that in healthy controls, besides, the -181G allele (A/G + G/G) genotypes significantly increased susceptibility to NSCLC compared with the A/A genotype, with OR=2.00[95% CI = 1.23-3.24] for NSCLC (137) (**Table 4**).However, in another study, MMP-7 -181 A/G polymorphism was found not significantly associated with LC susceptibility (138) (**Table 4**). Stratification analysis in this study showed that smoking did not significantly influence the association between the MMP-7-181A/G and NSCLC, and MMP-7-181A/G polymorphism was not associated with lymphatic metastasis in NSCLC patients, indicating that the MMP-7-181A/G polymorphism might not predict lymphatic metastasis in NSCLC (137). In conclusion, G allele might be risk allele. Besides, the results of these studies are confusing, and need to be further investigated in larger size of case–control studies.

Multivariate analysis revealed that MMP8 polymorphisms are not independent prognostic factors for overall survival (139). The polymorphisms MMP-8 -799 C/T may not play a major role in susceptibility to LC in Taiwanese (140) (**Table 4**). However, the 17 C/G polymorphism in MMP8 was associated with a statistically significant decreased risk of developing LC (OR= 0.65[95%CI = 0.45-0.93]) (139) (**Table 4**).

A meta-analysis suggests that the MMP9 -1562 C/T polymorphisms were risk factors for LC among Asians (141) (**Table 4**). However, the MMP9 -1562 T/T genotype was associated with a statistically significant decreased risk of developing LC, but no association was found between MMP9 -1562 C/T polymorphisms and risk of LC (133, 135, 147) (**Table 4**). Subgroup analysis by smoking status showed no association between the MMP-9 1562 C/T polymorphism and the risk of NSCLC. Furthermore, the genotype distribution between NSCLC patients with and without lymphatic metastasis was not significantly different. Therefore, MMP-9 1562 C/T polymorphism may not be used as a useful marker to predicate susceptibility and lymphatic metastasis in NSCLC (150). However, a meta-analysis suggested that MMP9 -1562 C/T might be protective factors (136) (**Table 4**). Meanwhile, MMP9-1562 C/T polymorphism was proved decreasing LC risk in Caucasians (131) (**Table 4**). All in all, T allele might be protective factor, and ethnicity is an important factor affecting LC development. Importantly, the results of these studies are conflicting, and need to be further investigated in larger size of case–control studies.

The genotypes of MMP-11 play a minor role in determining LC risk in Taiwanese. MMP-11 rs738791 T allele did not confer LC risk compared with the C allele. Besides, there was no association between rs2267029, rs738792 or rs28382575 and LC risk (142) (**Table 4**).

A study conducted in 2014 Caucasian LC patients and 1323 healthy controls showed that the G allele of the MMP-12 1082 A/G polymorphism was associated with higher risk of LC in men (OR=1.51[95%CI=1.04–2.09]; A/G + G/G versus A/A), but not in women, indicating G allele was risk allele (134) (**Table 4**). No association was found between the MMP-12 -82A/G polymorphism and risk of LC (134) (**Table 4**). Patients with stage I NSCLC carrying the variant G allele of the MMP-12 1082A/G polymorphism have worse OS and RFS, while MMP-12 −82A/G was not significantly associated with survival (145). Together, the G allele might be risk factor in men, and gender might be an important factor affecting LC susceptibility. Importantly, more analyses of case-control studies need to be done to figure out the results.

The MMP-13 77 A/G polymorphisms AG and GG genotypes significantly increased in lung tumor DNA compared with healthy controls (138). However, studies showed that the MMP13 -77A/G polymorphism was not associated with LC risk (139, 141) (**Table 4**). Besides, a study showed that the AA genotype was associated with a decreased risk of NSCLC compared with the GG genotype (143) (**Table 4**). Interestingly, a meta-analysis even suggested that MMP13 -77A/G might be protective factors among Asian (136) (**Table 4**). Together, MMP13 -77A/G might to some extent be protective factor. However, more analyses of case-control studies need to be done to further investigate the results.

## Discussion

In summary, roles of MMPs in LC are multi-faced. Most of MMPs are highly activated in cancers and contributed to cell proliferation and cancer development (8, 151–153). High expression of MMPs was mainly related to poor survival, high clinical stages and cancer metastasis. MMPs functions in a lot of physiological and pathological conditions. Expression of MMPs exerted significant prognostic value and predicated tumor recurrence in patients with LC. Besides, their expressions might be closely related with early-stage tumor invasion, metastasis, and angiogenesis, indicating their potential roles on monitoring progression and predicting prognosis of NSCLC. In addition, inhibition of MMPs might be useful for restoring sensitivity of LC cells to platinum, radiation, and immune therapy. Interestingly, MMPs polymorphisms also increase risk of LC. This review is the first to systematically clarify MMPs in LC. Although much work has been done, the reasons for increased MMP polymorphisms in LC, the mechanisms of MMPs induced CSCLs and the function of MMPs in tumor immunity should be further investigated. Accumulated evidences have shown that most of the MMP polymorphisms enhanced LC risks, but almost no study investigate the methods to decrease these risks, therefore, studies should be further explored to figure out the solutions. Nowadays, immunotherapy such as antibody‐mediated immune checkpoint blockade for LC has achieved great progress, however, LC remains the number one of cancer mortality (154, 155), indicating the necessities to uncover more new targets for immunotherapy. MMPs are closely involved in tumor immunity, significantly affected innate immune response. Targeting MMPs might be promising alternative to restore the host’s anti-tumor immune response and fight against LC cells.

## Author contributions

CW: writing, editing, and revising the manuscript.
